# Supplementary material for: Brain connectivity correlates of breathing and cardiac patterns in epilepsy: A study including SUDEP cases
Source: Imaging Neurosci (Camb). 2025 Oct 9;3:IMAG.a.918. doi: 10.1162/IMAG.a.918 (PMC12511790; doi:10.1162/IMAG.a.918)
Supplement: Supplementary Material [file IMAG.a.918_supp.pdf]

Supplementary Material

Group	SUDEP (n=9)	High-risk (n=46)	Low-risk (n=43)	Healthy controls (n=25)
Age (mean years ± SD)	26.2 ± 6.2	33.3 ± 9.6	29.5 ± 8.3	29.1 ± 6.1
Sex (male/female)	4/5	22/24	21/22	17/8
Age at epilepsy onset (years)	9.9 ± 6.8	10.0 ± 6.0	10.3 ± 7.3	-
Disease duration (mean years ± SD)	16.3 ± 9.6	23.2 ± 11.8	18.7 ± 9.7	-
TCS per month (mean ± SD)	7.7 ± 9.3	5.9 ± 7.4	N/A	-
Number of anti-seizure drugs (mean ± SD)	2.7 ± 0.7	2.8 ± 0.9	2.6 ± 1.1	-
Number of patients on polytherapy	9 (100%)	41 (89%)	34 (80 %)	-
**Epileptogenic hemisphere:**				
Left	5	22	21	-
Right	2	13	16	-
Non-lateralized	1	3	2	-
Unknown	1	8	4	-
**Epileptogenic zone:**				
Genealize	1	3	1	-
Multifocal	1	2	1	-
Hemispheric	1	2	2	-
Temporal	1	15	21	-
Occipital	1	2	0	-
Frontal	2	11	11	-
Temporo-occipital	0	0	1	-
Parietal	0	2	3	-
Parieto-occipital	0	0	1	-
Fronto-temporal	1	3	2	-
Insula	0	0	0	-
Putament	1	0	0	-
Unknown	0	6	0	-

Suppl. Table 1. Exp. 1. Group demographic and clinical summaries of epilepsy patients and healthy controls. SD=standard deviation, N.A.=not applicable, TCS=tonic-clonic seizures.

Supplementary Methods

a) Experiment 1. Preprocessing of the Human Connectome Project (HCP) dataset

We used resting-state fMRI scans from the HCP S1200 release (Van Essen et al., 2013) to examine the association of global signal amplitude (GSA) with variations in physiological variables. The HCP dataset includes, among others, T1-weighted (T1w) images and resting-state fMRI data (eyes-open and fixation on a cross-hair) from healthy young individuals (age range: 22-35 years) acquired on two different days. On each day, two 15-minute scans were collected (TR = 0.72 s). The preprocessing of the fMRI dataset is described in detail in Glasser et al. (2013). In the present study, the first scan from Day 1 was considered from 400 subjects who had good quality photoplethysmograph (PPG) and respiratory signal, as assessed by visual inspection.

The timings of the peaks in PPG were used to derive the heart rate, while the amplitudes of the PPG peaks were used to model the low-frequency variations in the envelope of the PPG signal. As described previously (Kassinopoulos & Mitsis, 2019, 2021), to facilitate peak detection, the PPG signal was initially band-pass filtered with a 2nd order Butterworth filter between 0.3 and 10 Hz. The minimum peak distance specified for peak detection varied between 0.5 and 0.9 s, depending on the subject's average heart rate. The heart rate signal was computed in beats-per-minute (bpm) and evenly resampled at 10 Hz. The amplitudes of the peaks were also evenly resampled at 10 Hz. The resampling of heart rate and PPG-Amp was done using linear interpolation. The breathing signal was detrended linearly and corrected for outliers using a median filter. Subsequently, the breathing signal was low-pass filtered at 5 Hz with a 2nd order Butterworth filter and z-scored. To extract the fMRI global signal of each scan, we initially performed tissue segmentation on the T1w images in the MNI152 space using FLIRT in FSL 5.0.9, which generated probabilistic maps for the grey matter, white matter and cerebrospinal fluid compartments (Zhang et al., 2001). Afterwards, the global signal was calculated by estimating the mean time-series across all voxels with a probability of belonging to GM above 0.25.

b) Experiment 2. Preprocessing of electrocardiogram (ECG)

The ECG was corrected for gradient artifacts using adaptive template subtraction (Allen et al., 2000) implemented in BrainVision Analyzer 2 software (Brain Products GmbH, Munich, Germany), and band-pass filtered from 0.5 to 40 Hz. The R-wave peaks were detected using Matlab's function *findpeaks* with a minimum peak distance varying between 0.5 and 0.9 s depending on the subject's average RR interval (time between successive R-waves).

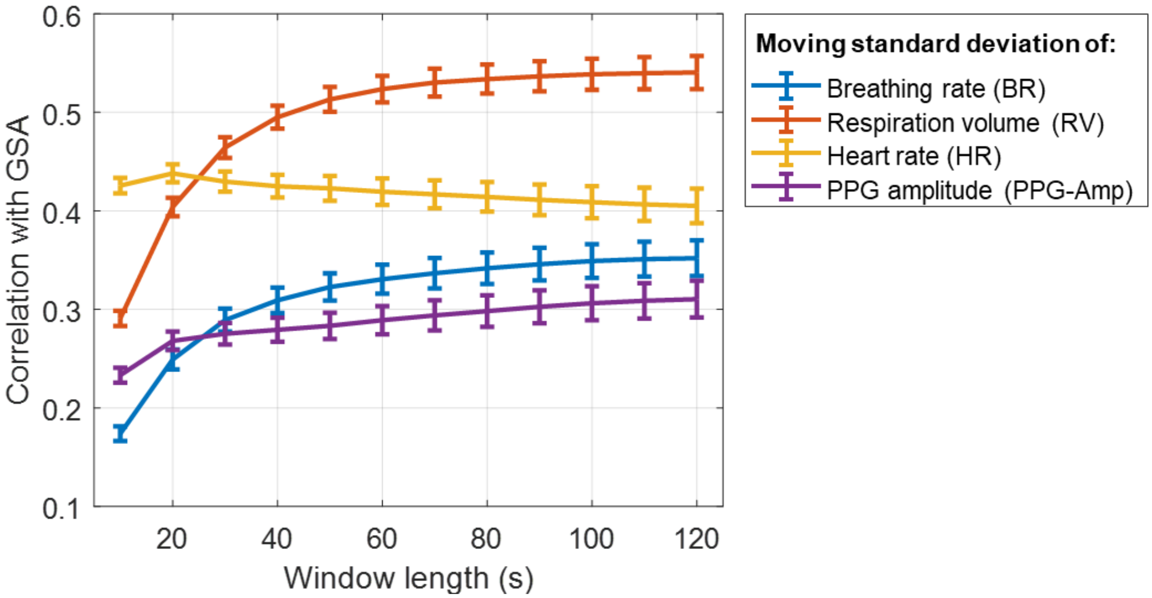

Suppl. Fig. 1. Exp. 1. Correlation of global signal amplitude (GSA) with the moving standard deviation of physiological variables for different window lengths. Resting-state fMRI data and concurrent photoplethysmograph (PPG) and breathing recordings from 400 healthy young subjects of the Human Connectome Project (Van Essen et al. 2013) were used for this analysis. The moving standard deviations of breathing rate, respiration volume, heart rate and PPG amplitude were computed for window lengths ranging from 10 to 120 sec in 10-second increments. Similarly, GSA (i.e. moving standard deviation of fMRI global signal) was computed for the same window lengths, and its correlation with the traces of the physiological variables was estimated and averaged across 400 subjects (error bars indicate standard errors). Increasing the window length from 20 to 80 sec was found to enhance the correlation of GSA with the amplitude of variations in breathing rate, respiration volume and PPG amplitude, whereas no substantial additional benefit was observed for longer lengths.

Suppl. Fig. 2 (Next page). Exp 1. Association of GSA with variations in (A) breathing rate, (B) heart rate and (C) PPG amplitude, during rest. In each panel, the first row shows the raw physiological signal (respiration or PPG), the second row shows the physiological variable extracted from the raw signal (i.e. breathing rate, heart rate or PPG amplitude), and the third row shows in black color the moving standard deviation of the physiological variables (window length: 80 sec). Overall, we observe that an increase in the levels of GSA can be induced by a transient apnea (HCP445535), a transient increase in heart rate (HCP495255) or strong fluctuations in PPG amplitude (HCP102008).

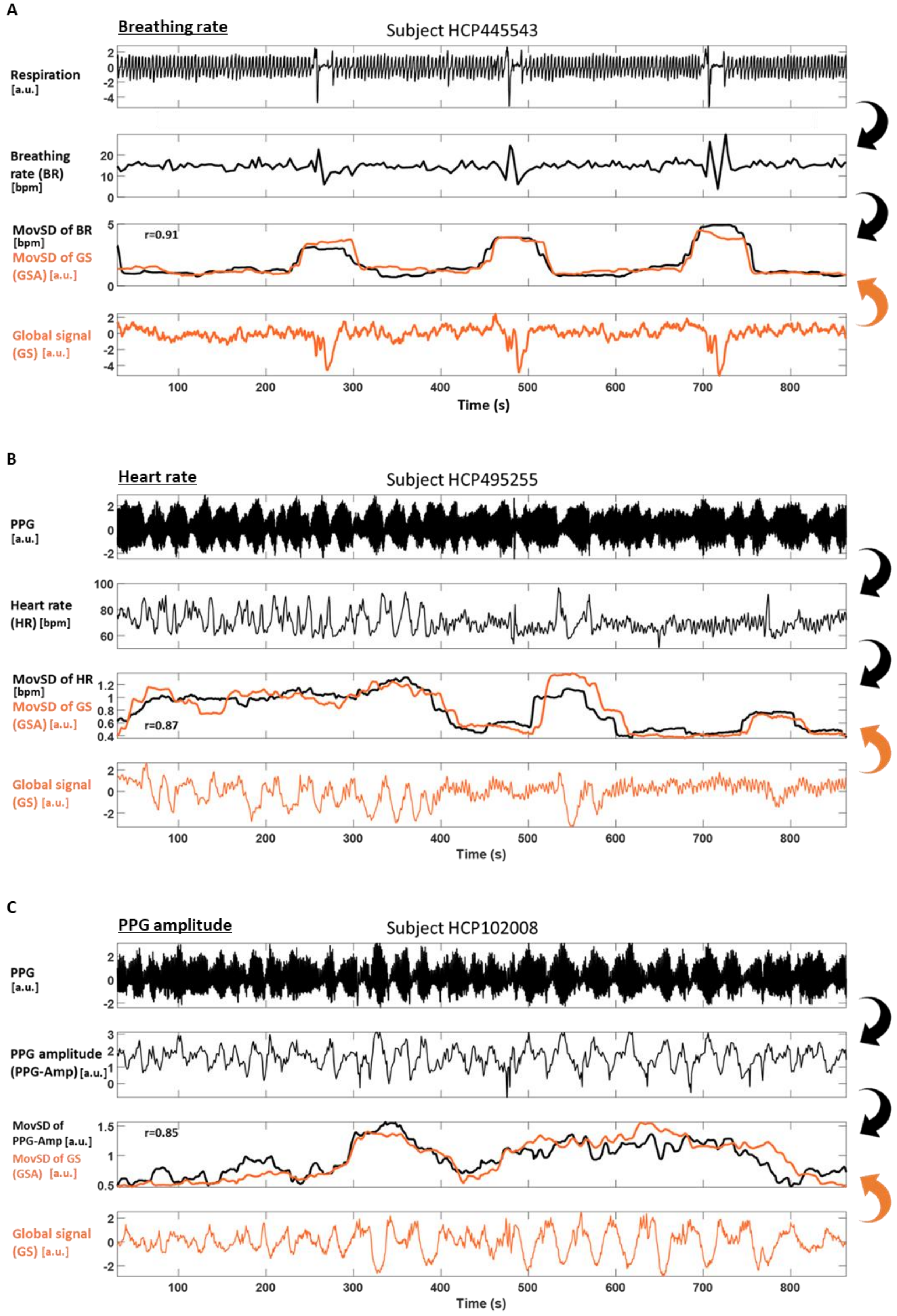
A
Breathing rate
Subject HCP445543
Respiration [a.u.]
Breathing rate (BR) [bpm]
MovSD of BR [bpm]
MovSD of GS (GSA) [a.u.]
r=0.91
Global signal (GS) [a.u.]
Time (s)
B
Heart rate
Subject HCP495255
PPG [a.u.]
Heart rate (HR) [bpm]
MovSD of HR [bpm]
MovSD of GS (GSA) [a.u.]
r=0.87
Global signal (GS) [a.u.]
Time (s)
C
PPG amplitude
Subject HCP102008
PPG [a.u.]
PPG amplitude (PPG-Amp) [a.u.]
MovSD of PPG-Amp [a.u.]
MovSD of GS (GSA) [a.u.]
r=0.85
Global signal (GS) [a.u.]
Time (s)

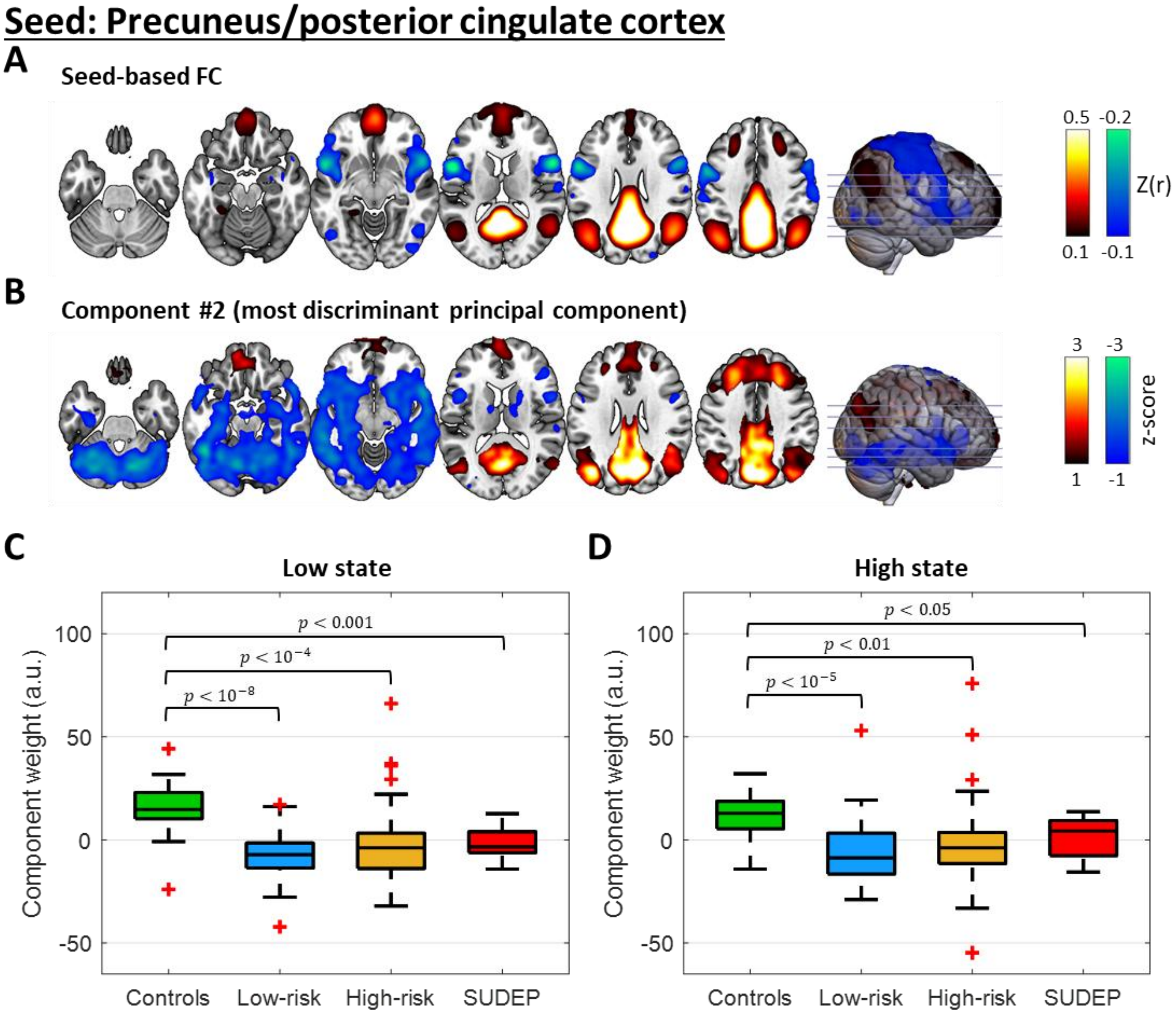

Suppl. Fig. 3. Exp. 2. Involvement of FC pattern #2 of precuneus/posterior cingulate (PCu/PCC) connectivity in the low and high state. (A) Seed-based correlation map with the seed placed in the PCu/PCC averaged across subjects and time. **(B)** FC pattern of component #2 derived from the PCu/PCC connectivity profiles of all subjects through PCA. Component weights of the four groups in the **(C)** low and **(D)** high state. For the spatial involvement of Yeo large-scale networks of FC pattern #2, see Suppl. Fig. 5.

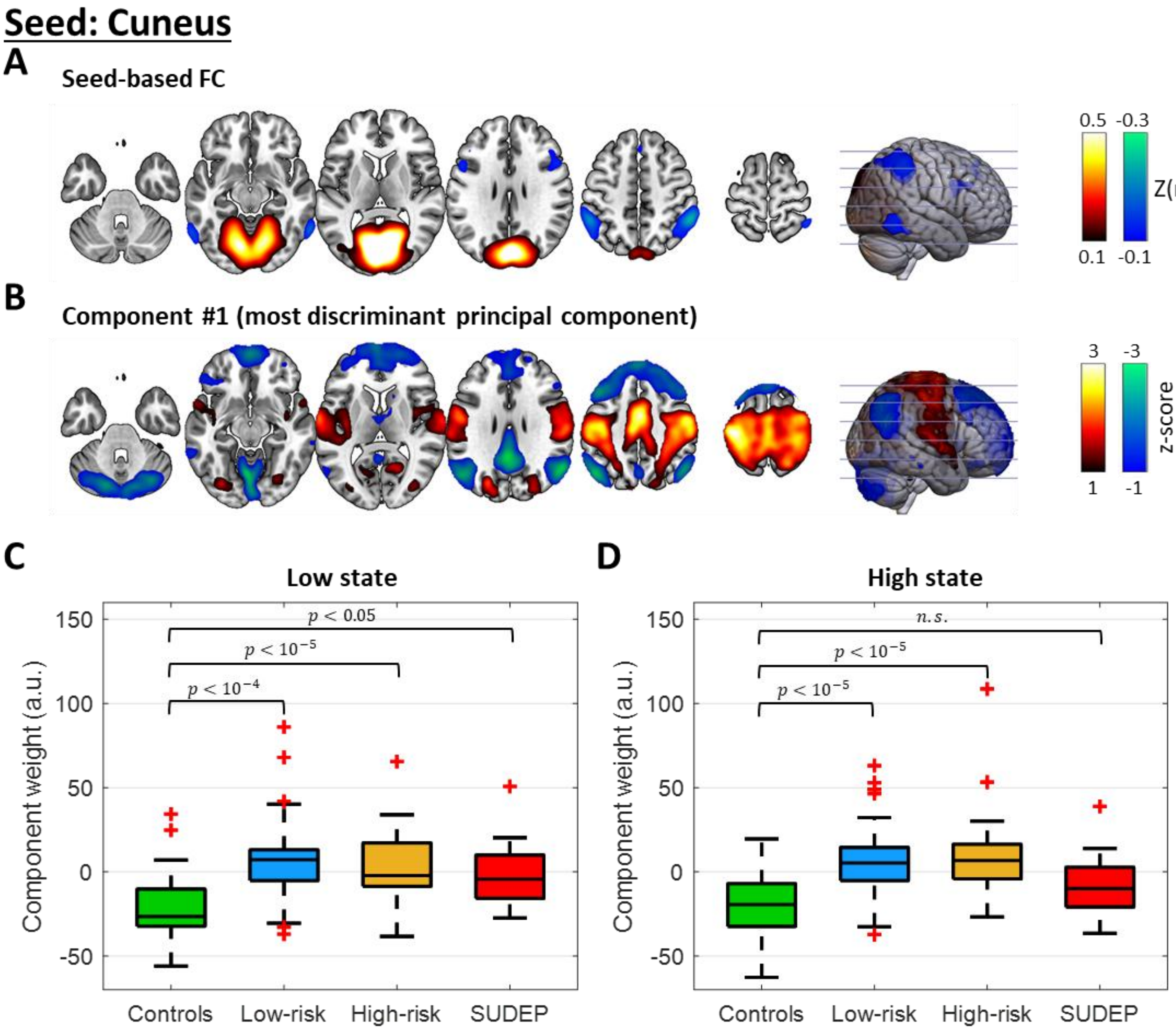

Suppl. Fig. 4. Exp. 2. Involvement of FC pattern #1 of cuneus in the low and high state. (A) Seed-based correlation map with the seed placed in the cuneus averaged across subjects and time. **(B)** FC pattern of component #1 from the cuneus connectivity profiles of all subjects using PCA. Component weights of the four groups in the **(C)** low and **(D)** high state. For the spatial involvement of the Yeo large-scale networks of FC pattern #1, see Suppl. Fig. 5.

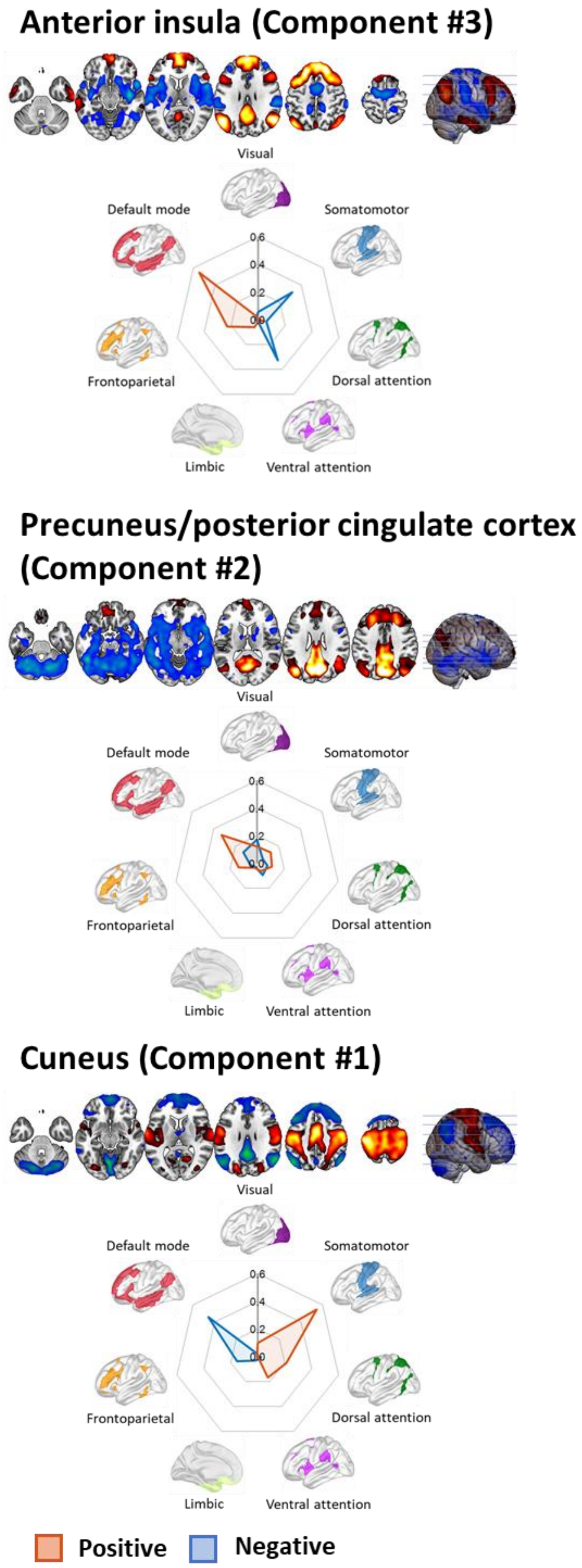

Suppl. Fig. 5. Exp. 2. Spatial involvement of the seven large-scale networks of the Yeo atlas (Yeo et al., 2011) in the FC patterns of the most discriminant components. The spatial involvement with the networks was assessed by calculating the Sørensen–Dice coefficient using the ICN_Atlas toolbox (Kozák et al., 2017) for the most discriminant components of the FC patterns, namely those that exhibited an F-statistic above chance level (F = 9.4, $p < 10^{-4}$; Fig. 3) for the anterior insula (top), PCu/PCC (middle) and cuneus (bottom). The sign of the components' constituent regions is represented in the involvement plots as red for positive and blue for negative.

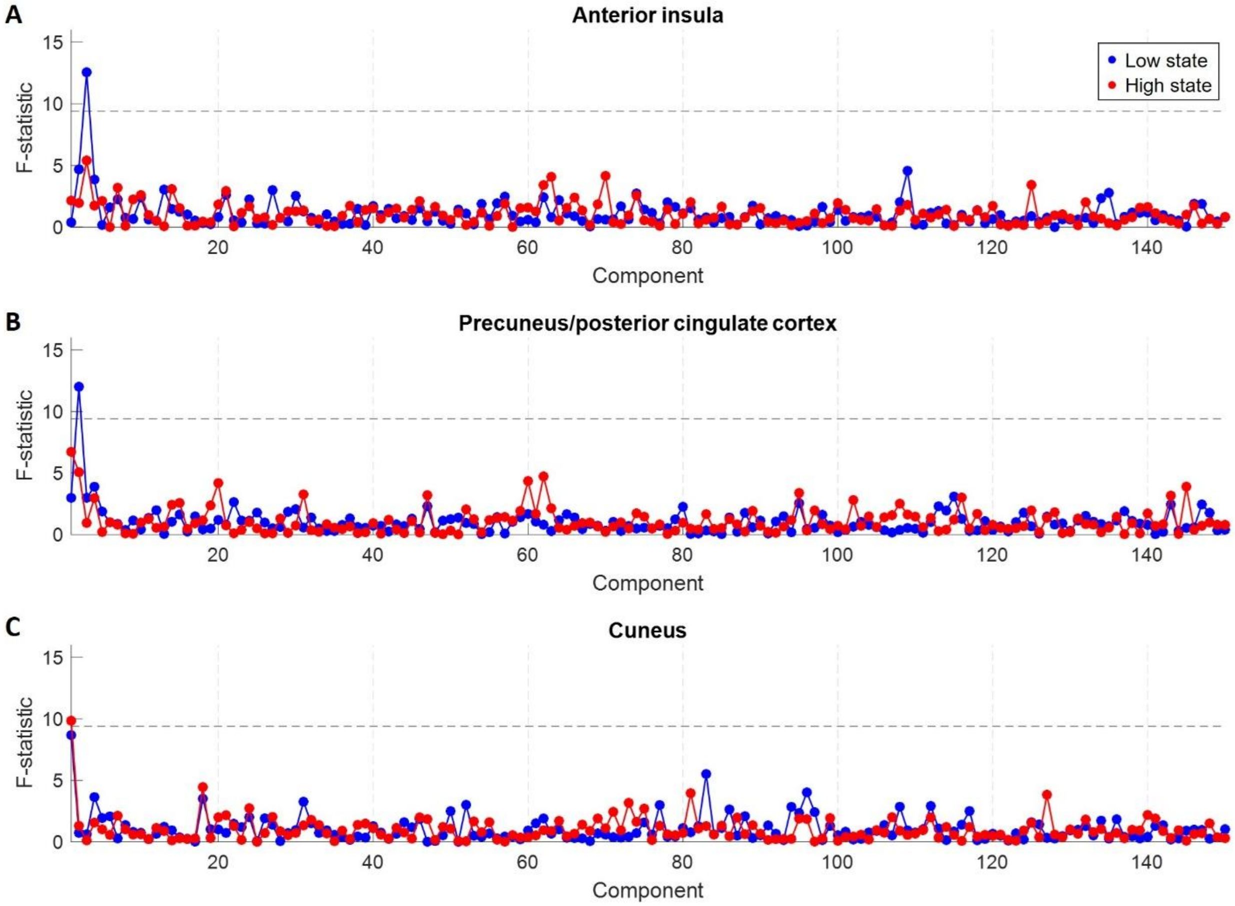

Suppl. Fig. 6. Exp. 2. *F*-statistics for assessing dispersion between groups based on FC patterns of (A) anterior insula, (B) precuneus/posterior cingulate cortex (PCu/PCC) and (C) cuneus, based on an analysis excluding the two 10-min fMRI scans. The dashed line indicates the chance level ($p < 0.05$, Bonferroni corrected), as determined by permutation distribution. All three regions had one of the first three principal components with an *F*-statistic at above chance level. None of the other nine seed regions examined here was found to yield a significant component in terms of *F*-statistic.

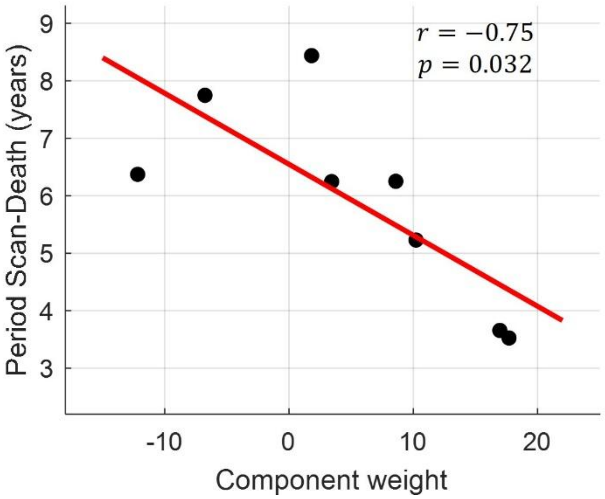

Suppl. Fig. 7. Exp. 2. Strength of anterior insula connectivity linked to the interval between the fMRI scan and time of SUDEP, based on an analysis excluding the two 10-min fMRI scans. The weight of component #3 in the high state (irregular cardiorespiratory activity) decreased with interval length ($r = -0.75$, $p = 0.032$).

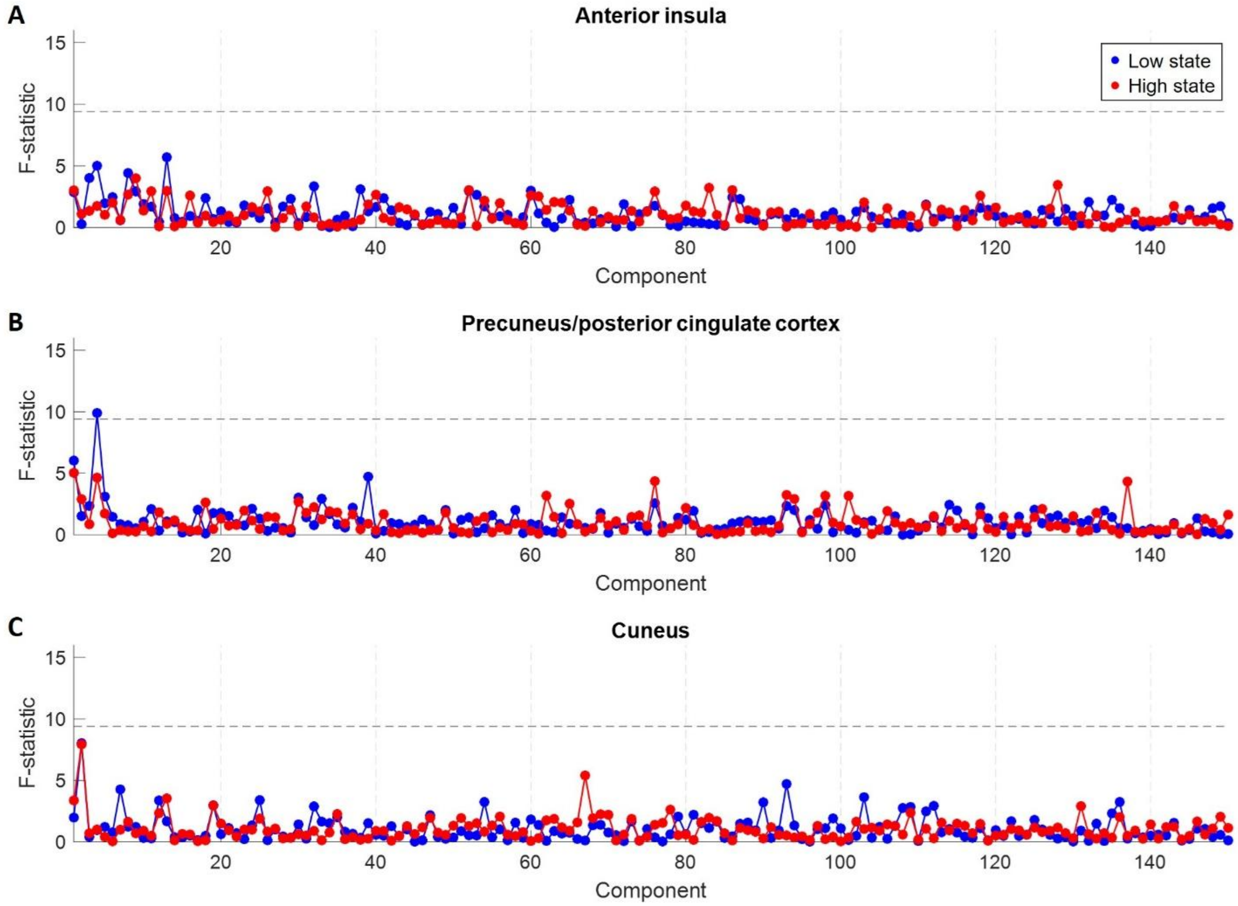

Suppl. Fig. 8. Exp. 2. *F*-statistics for assessing dispersion between groups based on FC patterns of (A) anterior insula, (B) precuneus/posterior cingulate cortex (PCu/PCC) and (C) cuneus, from an analysis omitting global signal regression (GSR). The dashed line indicates the chance level (p < 0.05, Bonferroni corrected), as determined by permutation distribution. Of the three regions that showed significant group discriminability in the main analysis (i.e., with GSR applied), only the PCu/PCC retained a component with an F-statistic exceeding the chance level when GSR was omitted. None of the remaining nine seed regions yielded significant components in this analysis.

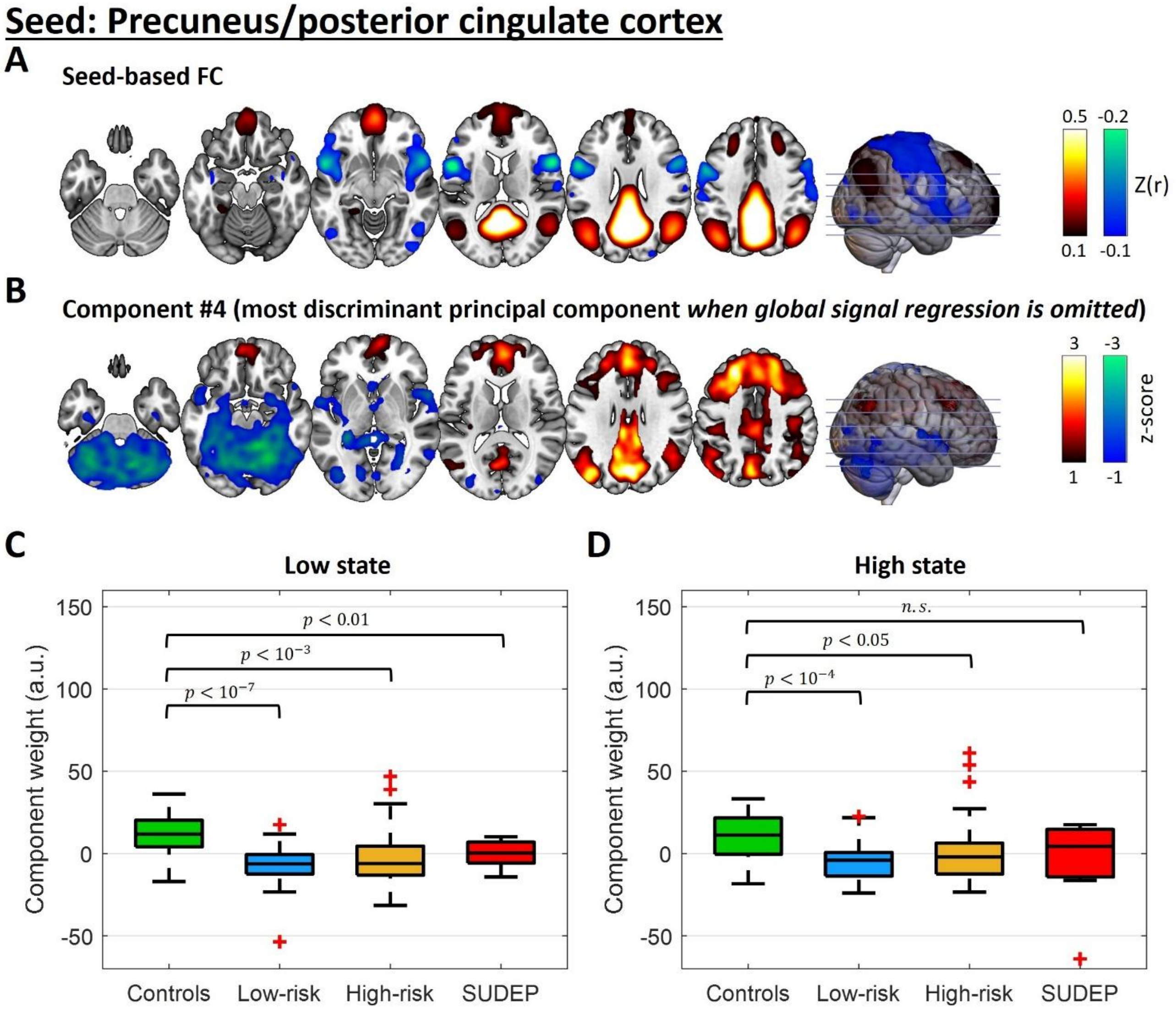

Suppl. Fig. 9. Exp. 2. Involvement of FC pattern #4 of precuneus/posterior cingulate cortex (PCu/PCC) in the low and high state, based on analysis omitting global signal regression (GSR). (A) Seed-based correlation map with the seed placed in the PCu/PCC, averaged across subjects and time. **(B)** FC pattern of component #4 derived from the PCu/PCC connectivity profiles of all subjects using PCA. Component weights of the four groups in the **(C)** low and **(D)** high state. n.s.: non-significant group differences.

References

Kassinopoulos, M., & Mitsis, G. D. (2019). Identification of physiological response functions to correct for fluctuations in resting-state fMRI related to heart rate and respiration. *NeuroImage*, *202*(September), 116150. https://doi.org/10.1016/j.neuroimage.2019.116150

Kassinopoulos, M., & Mitsis, G. D. (2021). Physiological noise modeling in fMRI based on the pulsatile component of photoplethysmograph. *NeuroImage*, *242*, 118467. https://doi.org/10.1016/j.neuroimage.2021.118467

Zhang, Y., Brady, M., & Smith, S. (2001). Segmentation of brain MR images through a hidden Markov random field model and the expectation-maximization algorithm. *IEEE Transactions on Medical Imaging*, *20*(1), 45–57. https://doi.org/10.1109/42.906424
